# ORESARA15 Acts Synergistically with ANGUSTIFOLIA3 and Separately from AINTEGUMENTA to Promote Cell Proliferation during Leaf Growth

**DOI:** 10.3390/ijms21010241

**Published:** 2019-12-29

**Authors:** Sang Eun Jun, Jin Hee Kim, Ji Young Hwang, Thien Tu Huynh Le, Gyung-Tae Kim

**Affiliations:** 1 Department of Molecular Genetics, Dong-A University, Busan 49315, Korea; junse033@hanmail.net (S.E.J.); hjyhjy123@naver.com (J.Y.H.); 2 Subtropical Horticulture Research Institute, Jeju National University, Jeju 63243, Korea; jhdragon@postech.ac.kr; 3 Department of Applied Bioscience, Graduate School of Natural Science, Dong-A University, Busan 49315, Korea; thientu07@gmail.com

**Keywords:** *AN3*, *ANT*, *Arabidopsis*, CELL proliferation, *ORE15*, *GRF*, *GIF*, leaf growth

## Abstract

Developing leaves undergo sequential coordinated cell proliferation and cell expansion to determine their final size and shape. Although several important regulators of cell proliferation have been reported, the gene network regulating leaf developmental processes remains unclear. Previously, we showed that ORESARA15 (ORE15) positively regulates the rate and duration of cell proliferation by promoting the expression of direct targets, *GROWTH-REGULATING FACTOR* (*GRF*) transcription factors, during leaf growth. In the current study, we examined the spatiotemporal patterns of *ORE15* expression and determined that *ORE15* expression partially overlapped with *AN3/GIF1* and *ANT* expression along the midvein in the proximal region of the leaf blade in young leaves. Genetic analysis revealed that ORE15 may function synergistically with AN3 to control leaf growth as a positive regulator of cell proliferation. Our molecular and genetic studies are the first to suggest the importance of functional redundancies between ORE15 and AN3, and between AN3 and ANT in cell proliferation regulatory pathway during leaf growth.

## 1. Introduction

Organ size is an important factor determining plant architecture and influencing adaptations to the environment. The final size of a plant organ is determined by two processes: Cell division and cell expansion. Cell proliferation is tightly regulated by many elements including genetic factors and phytohormones; however, the complete network of regulatory factors involved in cell proliferation during leaf development remains to be elucidated. Several studies have discovered individual cell cycle-related and cell proliferation regulatory factors, and their functional network has been summarized [1,2].

D-type *cyclins*, known as the *CYCD* family, are prime integrators for the cell cycle G1-to-S phase transition during organ growth. Overexpression of *CYCD3;1* induces ectopic cell division and inhibits cell expansion and endoreduplication [3,4]. CYCD3;1 is required for cell number control in developing organs by regulating the duration of the mitotic phase and the timing of the transition to the endocycle. Many studies have suggested that CYCD3;1 acts to determine cell cycle progression and maintain cell number balance. Another positive regulator, the AP2-type transcription factor *AINTEGUMENTA* (*ANT*), enhances cell proliferation by prolonging the duration of *CYCD3;1* expression and promoting an unknown cellular growth pathway, resulting in smaller organs and fewer cells in loss of function (LOF) mutants, whereas larger organs and more cells result from the period of extended growth in gain of function (GOF) mutants [5,6]. Overexpression of the novel auxin-inducible gene *ARGOS* has been shown to extend *ANT* and *CYCD3;1* expression, ultimately enlarging the plant. These results suggest that ARGOS regulates cell proliferation and leaf growth through the ANT–CYCD3;1 pathway [7]. However, CYCD3;1 activity is insufficient to influence organ growth [4], and combined mutation of members of the *CYCD3* family does not abolish organ growth [6]. The exact links between ANT and cellular growth remain unknown.

Another important cell proliferation regulator of organ growth, *ANGUSTIFOLIA3/GRF-INTERACTING FACTOR1* (*AN3/GIF1*), is a member of the transcriptional coactivator family in *Arabidopsis* [8,9]. AN3/GIF1 is recruited by DNA-binding factors such as GROWTH REGULATOR FACTORs (GRFs) to activate target gene expression [10,11]. Mutations in *GRF3*, *GRF4*, or *GRF5* have been shown to decrease cell proliferation activity, thereby reducing leaf size [12,13]. *GIF*s have been found to redundantly regulate leaf blade development through cell proliferation regulation [14,15,16], acting in a non-autonomous manner [17]. Recent studies have reported that the AN3/GIF1 protein recruits SWITCH/SUCROSE NONFEREMNTING (SWI/SNF) chromatin remodeling complexes to regulate leaf development [17,18,19].

The *TCP* (*TEOSINTE BRANCHED 1*, *CYCLOIDEA*, and *PROLIFERATING CELL FACTORS 1 AND 2*) family members have also been identified as cell proliferation regulators in meristems and organ primordia [20]. Recent findings have revealed a second class of *TCPs*, whose expression is regulated by *miRNA319*, which plays important roles in multiple developmental processes including leaf growth, morphogenesis, and senescence [21,22,23]. TCP4 affects cell proliferation regulation via the induction of *miRNA396*, which represses the *GRF* family, and by direct connection with KIP-RELATED PROTEIN 1 (KRP1) which terminates the cell cycle and induces the transition from mitotic cell cycle to the endocycle causing an increase in DNA ploidy by directly interacting with cell cycle genes [2,24]. Previously, we isolated and characterized *ORESARA 15* (*ORE15*) encoding a plant A/T-rich sequence-and zinc-binding protein (PLATZ) family transcription factor and determined that *ORE15* enhances leaf growth by promoting the rate and duration of cell proliferation in its early stage and suppresses leaf senescence in its late stage by modulating the *miRNA396*–GRF–GIF regulatory pathway [25]. It has been proposed that ORE15 may act as a transcriptional activator since it positively regulates the expression of *GRF1* and *GRF4* through direct binding to their promoters [25]. Together, these results suggest that ORE15 may work together with GRF–GIF to control the cell proliferation in leaf growth.

Although several important regulators involved in the control of cell proliferation have been reported, the molecular basis of the regulatory gene network during leaf developmental processes remains unclear. In this study, our characterization of cell proliferation during leaf growth using LOF and GOF mutations of *ORE15* suggests that *ORE15* positively regulates cell proliferation during leaf growth. We also generated double mutants of cell proliferation-related genes, *ANT*, and *AN3/GIF1* using a combination of LOF and GOF *ORE15* mutants and performed genetic and anatomical analyses to explore the relationship among ORE15, AN3/GIF1, and ANT in cell proliferation regulatory pathways during leaf growth.

## 2. Results

### 2.1. ORE15 Is Expressed in the Proximal Region of the Leaf Blade and in Petioles of Young Leaves

**Figure 1:**
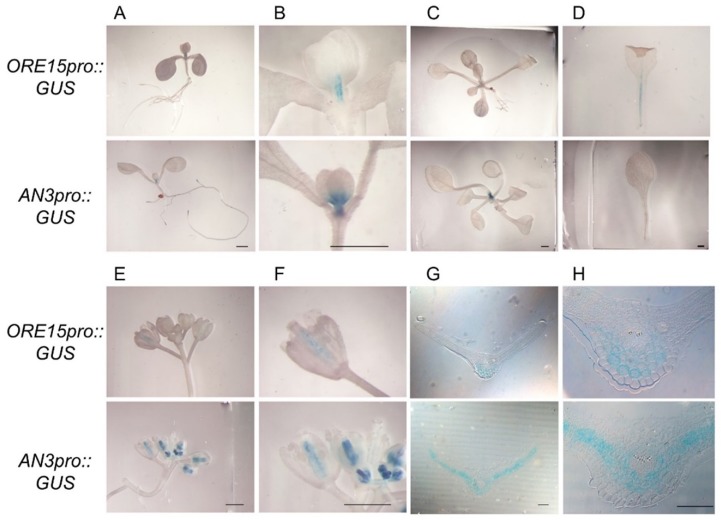
Spatial and temporal specific expression of *ORE15* and *AN3* determined by promoter::β-glucuronidase (GUS) assay of transgenic plants harboring the *ORE15*promoter::*GUS* and *AN3*promoter::*GUS* construct. (**A**) Seedling with the first pair of true leaves at seven days after sowing (DAS). (**B**) Magnified view of (A). (**C**) Seedling at 14 DAS. (**D**) First true leaf pair in the juvenile phase at 14 DAS. (**E**) Young floral bud. (**F**) Mature flower. Scale bar = 10 mm. (**G**) Transverse section of the proximal part of leaf blades from GUS-stained third leaf at 14 DAS. (**H**) Magnified view of (G). Scale bar = 100 μm.

Our previous study demonstrated that ORE15 enhances leaf growth by promoting cell proliferation in the early stage of leaf development by modulating the GRF–GIF [25]. ORE15 also bound directly to the promoters of the *GRF1* and *GRF4* genes, which play roles in cell proliferation in leaf primordia [8,9,25], but did not bind directly to *ANT* and *AN3/GIF1* promoter. To further explore ORE15 function in organ growth, we determined the temporal and spatial expression patterns of *ORE15* using a promoter::β-glucuronidase (GUS) approach. *ORE15* was expressed in the proximal part of the leaf blade and in leaf petioles (Figure 1A–D). Strong *ORE15* expression was observed mainly along the midveins of young leaves (i.e., the first to fifth leaves) produced at 14 days after sowing (DAS) and throughout the petiole (Figure 1A–D). Similar spatial expression of *ORE15* along the midvein was observed during the cell proliferation stage in leaves (Figure 1A–D). However, *ORE15* expression was not detected either in shoot apical meristem or leaf primordia (Figure 1A–C). *ORE15* expression was detected in the carpels of mature flowers (Figure 1E,F), but not in young flowers, mature leaves, or roots. Our expression analysis of ORE15 supported previous findings obtained by reverse transcription polymerase chain reaction (RT-PCR) gene expression analysis [25].

*AN3/GIF1* was strongly expressed in the proximal region of the leaf during the cell proliferation stage (Figure 1A–C), which is consistent with previous findings [9]. *AN3* was also expressed in the carpels of both young and mature flowers (Figure 1E,F). *ANT*, another important regulator of cell proliferation, has been reported to be expressed in active cell proliferation regions including young leaf blades, veins of leaves and stems, pistils, and meristems [26]. A study of *GRF1* and *GRF4,* which were direct downstream targets of *ORE15* [25], also showed very similar expression patterns to those of *ORE15* [9,27]. To compare the expression domains of *ORE15* and *AN3/GIF1* in detail, we conducted histochemical analysis of GUS-stained leaves of transgenic plants. An examination of transverse sections of cell proliferating leaves of *ORE15*promoter::*GUS* transgenic plants showed that *ORE15* was expressed strongly in the abaxial parenchyma cells around midvein in the proximal region of the leaf blade (Figure 1B,D,G,H). Transverse sections of GUS-stained leaves from *AN3*promoter::*GUS* transgenic plants showed that *AN3* was expressed in mesophyll cells in the proximal region of the leaf blade of young leaves (Figure 1G,H), which was consistent with a previous report [17]. These results indicate that spatiotemporal expression patterns of *ORE15* partially overlap with those of *AN3/GIF1* in the abaxial parenchyma cells around midvein in the proximal region of the leaf blade. Together, these expression findings supported the idea of genetic interaction between the ORE15 and GRF–GIF complex, especially AN3/GIF1, GRF1, and GRF4 in the regulation of cell proliferation.

### 2.2. ORE15 Acts Synergically with AN3 and Separately from ANT to Promote Leaf Growth

In a previous study, we elucidated the regulatory relationship between ORE15 and the GRF–GIF regulatory module at the genetic level. The results of our expression study indicated that ANT expression was downregulated by ORE15 [25].

**Figure 2:**
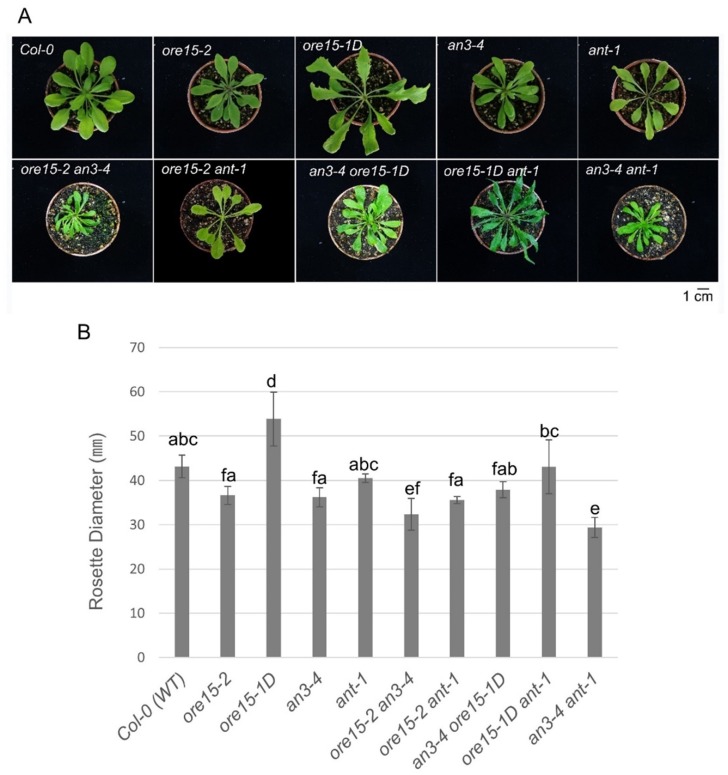
Plant morphology and rosette diameter of the *Arabidopsis* Columbia-0 (Col-0) wild type (WT), single loss of function (LOF) and gain of function (GOF) mutants. (**A**) Plant phenotypes of the *ore15-2*, *ore15-1D*, *an3-4*, *ant-1*, and combined double mutants, *ore15-2 an3-4*, *ore15-2 ant-1*, *an3-4 ore15-1D*, *ore15-1D ant-1*, and *an3-4 ant-1* at 35 DAS. *ore15-2* (SALK_029507) is a T-DNA insertional mutant and *ore15-1D* is a dominant mutant isolated from activation tagging lines, in which T-DNA was inserted in the first intron and 3′ UTR of *ORE15*, respectively. *an3-4* has a large deletion in the *AN3* locus and *ant-1* has a 22-nucleotide deletion in the second exon of the *ANT* locus. Scale bar = 1 cm. (**B**) The widest diameter of rosette leaves. Data are means ± standard error (SE) (*n* = 5). Means were compared using one-way analysis of variance (ANOVA) followed by Duncan’s multiple range test. Different lowercase letters indicate significantly different means between genotypes.

**Figure 3:**
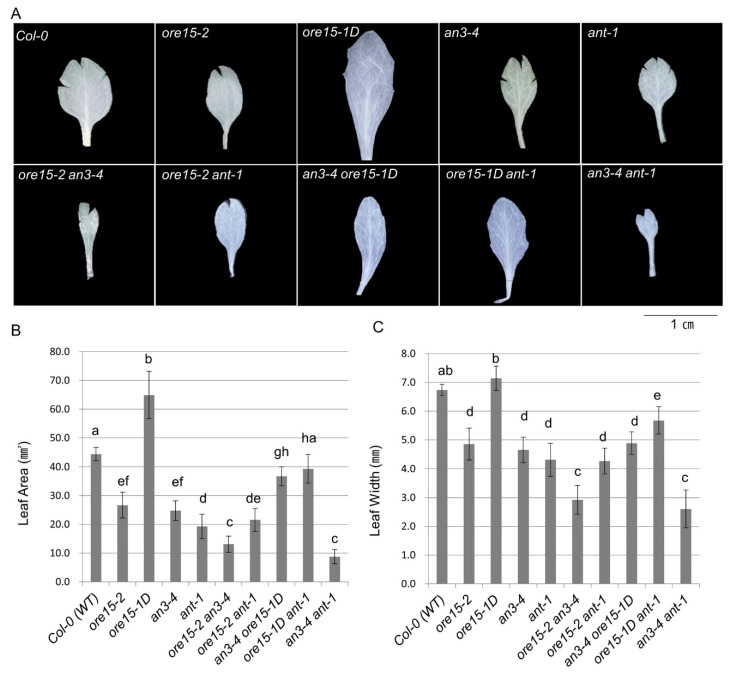
Leaf morphology of the mature third leaf from Col-0 (WT), single mutant, and double mutant plants at 21 DAS. (**A**) Phenotype of the mature third leaf detached from Col-0, single mutant, and double mutant plants at 21 DAS. Scale bar = 1 cm. (**B**) Leaf area. (**C**) Leaf width. Data are means ± standard error (SE) (7 ≤ *n* ≤ 10). Means were compared using one-way analysis of variance (ANOVA) followed by Duncan’s multiple range test. Different lowercase letters indicate significantly different means between genotypes.

**Figure 4:**
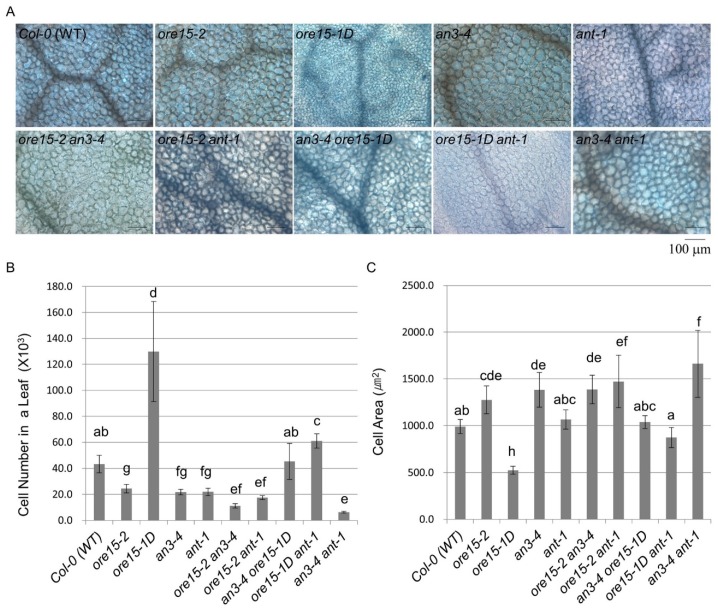
Statistical analysis of leaf palisade mesophyll cell number and area in mature third leaves from Col-0 (WT), single mutant, and double mutant plants at 21 DAS. (**A**) Paradermal images of palisade mesophyll cells on mature third leaves. Scale bar = 100 µm. (**B**) Cell number. (**C**) Cell area. Data are means ± SE (4 ≤ *n* ≤ 7). Means were compared using one-way ANOVA followed by Duncan’s multiple range test. Different lowercase letters indicate significantly different means between genotypes.

To identify the genetic relationship between ORE15, AN3, and ANT, we generated *ore15-2 an3-4*, *ore15-1D an3-4*, *ore15-2 ant-1*, and *ore15-1D ant-1* by crossing a combination of LOF and GOF mutants and measured mature third leaves in the resulting mutant lines. We found that *ore15-2 an3-4* plants had much smaller leaves and cell numbers than each single mutant (Figure 2, Figure 3 and Figure 4) [25]. *ore15-1D an3-4* plants showed median size of leaves compared with the two parental lines (Figure 2 and Figure 3). The number of palisade cells produced in leaves of the *ore15-1D an3-4* double mutant were 34.0%, 203.8%, and 102.2% of those produced in *ore15-1D*, *an3-4*, and Columbia-0 (Col-0) leaves, respectively (Figure 4), indicating the genetic interaction between ORE15 and AN3. Thus, ORE15 may function synergistically with AN3 to control leaf growth as a positive regulator of cell proliferation.

Both *ore15-2* and *ant-1* mutants produced smaller leaves than Col-0 (Figure 2 and Figure 3). The LOF double mutant *ore15-2 ant-1* produced slightly smaller and slightly lager leaf area than *ore15-2* and *ant-1* single mutant, respectively (Figure 3, Table S1). *ore15-1D ant-1* showed an intermediate phenotype of each single mutant, producing smaller and larger leaves than *ore15-1D* and *ant-1*, respectively (Figure 3). In addition, *ore15-1D ant-1* leaves showed serrated phenotype, the same as *ore15-1D* leaves (Figure 2A). These results suggest that ORE15 may act separately from the ANT pathway to promote leaf growth.

### 2.3. ORE15 Regulates Cell Proliferation during Leaf Growth Independently to ANT

Leaf growth is regulated by cell proliferation and cell expansion [28,29]. As previously reported, ORE15 promotes leaf growth by enhancing the rate and duration of cell proliferation. In our previous study, the *ore15-2 an3-4* double mutant produced significantly smaller leaves and cell numbers, indicating a composite regulatory interaction between ORE15 and AN3/GIF1 in the regulation of leaf growth [25].

To further elucidate a potential regulatory interaction between ORE15 and ANT, we determined the number and area of leaf cells in mature third leaves collected from GOF and LOF mutants of *ORE15* and *ANT* (Figure 4, Table S2). The numbers of palisade cells produced in leaves of the *ore15-2 ant-1* LOF double mutant were 71.9%, 80.3%, and 40.7% of those produced in *ore15-2*, *ant-1*, and Col-0 leaves, respectively (Figure 4 and Figure S1). The *ore15-1D ant-1* double mutant produced fewer cells (46.9%) than *ore15-1D* but more cells (278.1%) than *ant-1* (Figure 4 and Figure S1), indicating an independent gene action. Together, our results suggest that ORE15 may act as a positive regulator promoting cell proliferation, separately from the ANT pathway during leaf growth (Figure 6).

On the other hand, the *ore15-1D* mutant produced smaller cells than Col-0; however, *ore15-2* produced larger cells than Col-0 (Figure 4), indicating that ORE15 may negatively regulate cell area during leaf growth. Since endoreduplication is a determinant of cell expansion [30,31], we performed flow cytometric analysis of mature leaves of Col-0, *ore15-2*, and *ore15-1D*. Ploidy levels of *ore15-2* and *ore15-1D* were slightly higher and lower than that of Col-0 (Figure S2). These results indicate that ORE15 may be a minor factor influencing negative promotion of endoreduplication in leaves. The *ore15-2 ant-1* LOF double mutant produced cells that were 115.5% and 138.2% larger than those of *ore15-2* and *ant-1*, respectively (Figure 4). By contrast, *ore15-1D ant-1* produced the median cell area among each single mutant (Figure 4). These results indicate that ORE15 negatively regulates cell expansion, which may be a secondary effect of the ORE15- and ANT- cell proliferation pathway or a compensation process due to inhibition of cell division in the leaf organ [32].

### 2.4. AN3 Genetically Interacts with ANT to Promote Cell Proliferation during Leaf Growth

Our previous studies suggested that ORE15 plays a role in the AN3/GIF1-mediated cell proliferation regulatory pathway. The results of the current study indicate that ORE15 regulates cell proliferation during leaf growth, independently to ANT. To explore the molecular mechanism of cell proliferation in leaf growth, we analyzed the genetic interaction between AN3 and ANT using double mutants generated by crossing *an3-4* and *ant-1*. Interestingly, leaves were significantly smaller in the *an3-4 ant-1* LOF double mutant than in each single mutant (Figure 2 and Figure 3). These results indicate that AN3 and ANT synergistically interact with each other to control leaf growth.

To further elucidate the relationship between AN3 and ANT in the cell proliferation pathway, we examined the number and area of leaf cells in mature third leaves of double mutants (Figure 4). The *an3-4 ant-1* double mutant produced significantly fewer cells than each single mutant (29.2%, 28.9%) and Col-0 (14.6%) (Figure 4 and Figure S1). Cells produced by *an3-4 ant-1* were much larger than those of either *an3-4* or *ant-1* (Figure 4), under compensation process due to reduced cell division [32]. These results suggest that AN3 and ANT synergistically control cell proliferation, indicating that AN3 genetically interacts with ANT for regulating cell proliferation during leaf growth.

### 2.5. ORE15 Influences Cell Proliferation by Affecting Genes That Regulate Cell Division

**Figure 5:**
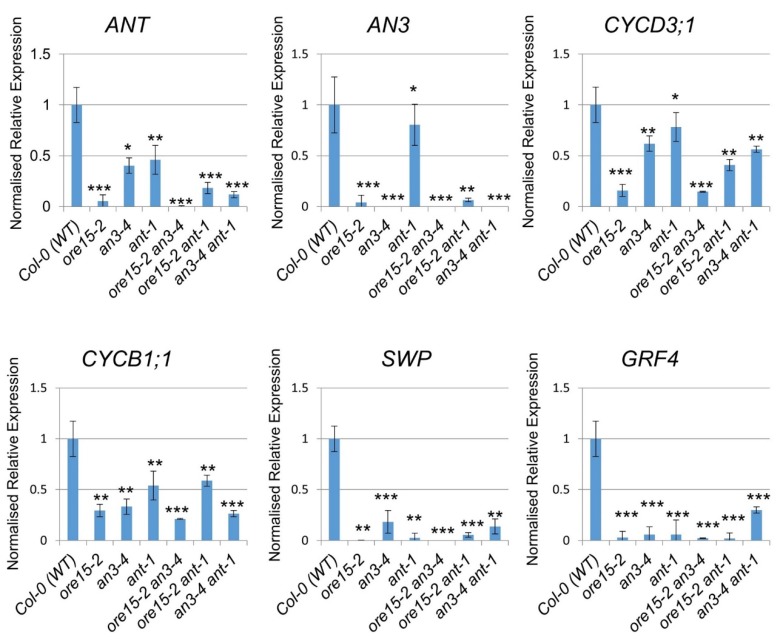
Expression of cell proliferation-related genes in the blades of cell proliferating leaves (third to fifth leaves) of Col-0 (WT), single mutant, and double mutant plants at 13 DAS. The transcript abundance of each gene was analyzed by quantitative reverse transcription polymerase chain reaction (qRT-PCR) using primers specific to each gene, normalized to *TUB4*, and are shown as values relative to each level of the Col-0 (WT). Data are representative of three technical replicates. Plotted values are means ± SE (*n* = 3). A statistical analysis was performed using a one-way ANOVA. Asterisks indicate significant difference from Col-0 (WT) plants (*, *p* < 0.1; **, *p* < 0.05; ***, *p* < 0.01).

To gain further insight into the molecular mechanisms by which AN3 and ANT mediate the cell proliferation regulatory pathways underlying leaf growth, we analyzed expression levels of several leaf growth factors controlling cell proliferation or cell division in the third leaf blade during the cell proliferation stage among LOF single and double mutants using quantitative RT-PCR (Figure 5). The expression of *AN3* and *ANT* was strongly reduced in *ore15-2* and all double mutants compared with Col-0 (Figure 5), supporting the idea of potential gene interaction among ORE15, AN3, and ANT. In addition, the expression of *ANT* in *an3-4* and that of *AN3* in *ant-1* was strongly reduced (Figure 5), also supporting our hypothesis of interaction between ANT and AN3.

*Cyclin B1;1* (*CYCB1;1*) expression was reduced in all mutants compared with Col-0, although differences were detected in the extent of reduction (Figure 5). *CYCD3;1* expression was greatly reduced in *ore15-2*, *ore15-2 ant-1*, *an3-4 ant-1*, and *ore15-2 an3-4* LOF double mutants, indicating that ORE15 and the ANT pathway coordinate to regulate cell proliferation. *GRF4* expression was also greatly reduced in all single and double mutants compared with Col-0 (Figure 5), supporting a relationship between ORE15 and the GRF–GIF. The expression of *STRUWWELPETER* (*SWP*), which encodes a protein similar to subunits of the mediator complex [33], was greatly reduced in all single and double mutants compared with Col-0 (Figure 5), indicating that SWP might have an important role in cell proliferation regulation during leaf growth.

## 3. Discussion

### 3.1. ORE15 May Act to Maintain AN3 and ANT Expression in the Later Stage of Cell Proliferation during Leaf Growth

In this study, the *ore15-1D* and *ore15-2* mutants produced larger and smaller leaves, respectively, than the Col-0 (Figure 2 and Figure 3), consistent with the findings of our previous study [25]. Kinematic analysis of leaf growth showed a higher rate of cell proliferation in *ore15-1D* in early stage of leaves compared with those in Col-0 [25]. Anatomical analysis also showed that ORE15 mediated the cell proliferating phase of early stage in leaf growth [25].

Our *ORE15*promoter::*GUS* analysis indicated that *ORE15* was expressed in the abaxial parenchyma cells along the midvein region of the proximal part of the leaf blade and throughout the leaf petiole (Figure 1). On the other hand, *AN3* was expressed in the adaxial and abaxial parenchyma cells along the midvein region of the proximal part of the leaf blade (Figure 1). In spite of the different spatial expression patterns in adaxial parenchyma cells between *ORE15* and *AN3*, two genes showed genetic interaction in cell proliferation. This discrepancy could be explained by non-cell autonomous regulation of AN3 [17] and the possibility of ORE15 action in non-cell autonomous manner. Further studies are required to clarify the detail of interaction between ORE15 and AN3 in leaf growth. In addition, the temporal expression pattern of *ORE15* partially overlapped with that of *AN3* (Figure 1) and *ANT*, as also shown previously [9,17,26]. *ORE15* expression was not detected in leaf primordia. On the other hand, strong *AN3* and *ANT* expressions were observed in the proximal region of leaf primordia and young leaves (Figure 1A–C) [9,17,26]. A chromatin immunoprecipitation (ChIP) study of *GRF1* and *GRF4* promoters, which were found to be putative targets of the *ORE15* gene in our previous study [25], showed very similar expression patterns to *ORE15* [9]. Moreover, *GRF1* and *GRF4* expression was weaker than *AN3* expression in the proximal region of leaf primordia [9]. These results indicated that ORE15 may function to maintain *AN3* and *ANT* expression in the later stage of cell proliferation for leaf growth. Together, our result of the spatiotemporally overlapping expression of ORE15 and the GIF–GRF complex, especially AN3/GIF1, GRF1, and GRF4, supports our working model of the cell proliferation regulation to promote leaf growth.

### 3.2. Functional Redendancy between ORE15 and AN3, and between AN3 and ANT in Cell Proliferation Regulatory Pathways during Leaf Growth

**Figure 6:**
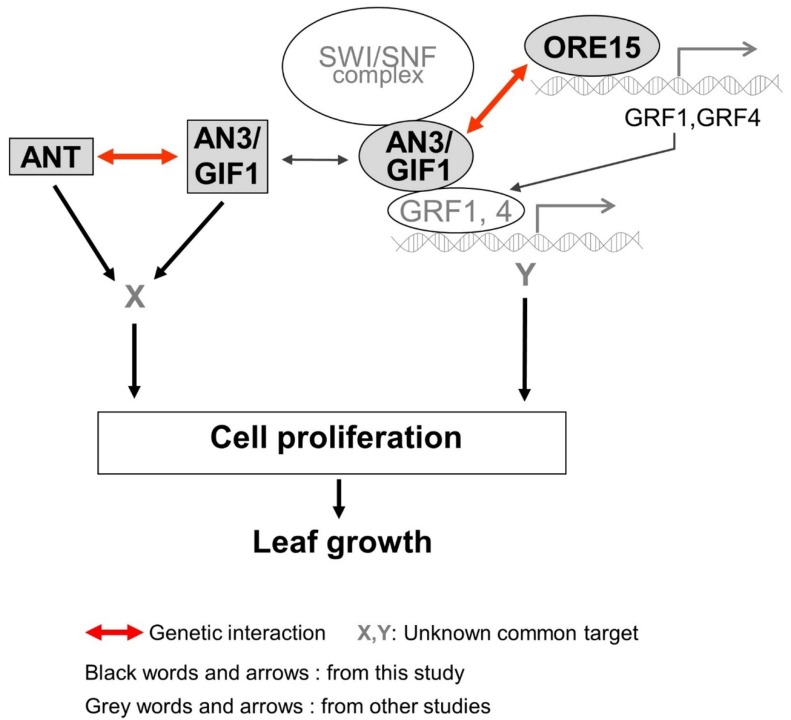
A working model of the genetic interaction between ORE15, AN3, and ANT for cell proliferation regulatory pathway during leaf growth. Two functionally redundant parallel pathways might converge on common nodes, by regulating unknown target X, Y genes.

Our previous study indicated that ORE15 enhances leaf growth by promoting cell proliferation in the early stage of leaf development by modulating the AN3/GIF1–GRF and delays leaf senescence at the mature stage of leaf development [25]. In this study, the more severe leaf size defect and more reduced cell number in leaves in *ore15-2 an3-4* double mutant (Figure 3 and Figure 4) suggest that there is some functional redundancy between ORE15 and AN3 and that these two proteins work in parallel pathways that promote leaf growth (Figure 6). These pathways might converge on a common node, perhaps by regulating some common target gene [34]. *ORE15* also enhances the transcription of *ANT*, *CycD3;1*, and *CycB1:1* (Figure 5), which are involved in promoting cell division activity [25]. The ARGOS–ANT–CYCD3 pathway is another well characterized pathway involved in cell proliferation-mediated leaf growth [5,7,35].

Therefore, we analyzed the relationship between *ORE15* and another important regulator, *ANT*. The double mutant *ore15-2 ant-1* had more reduced cell number in leaves than each single mutant (Figure 4), indicating that *ORE15* may act separately from *ANT* in the cell proliferation regulatory pathway (Figure 6). The serrated leaves in the *ore15-1D ant-1* mutant also support that ORE15 and ANT might work separately in leaf growth.

To date, the relationship between *ANT* and *AN3* for proper cell proliferation during leaf development has remained unknown. Recently, it was reported that *gif1/an3 ant-1* double mutant had small plants with fewer cells than its parental lines and displayed remarkable synergism in leaf development in supplementary data [16], indicating the possible genetic interaction between AN3/GIF1 and ANT. In the current study, the *an3-4 ant-1* double mutant produced narrower and smaller leaves than all single mutants (Figure 2 and Figure 3). In addition, the *an3-4 ant-1* double mutant produced significantly reduced cell number than all single mutants (Figure 4), indicating that these two genes may act synergistically as regulators of the cell proliferation pathway. However, the molecular mechanism of interaction between these two factors is still unclear. Together, these findings provide the first evidence to suggest that there is some functional redundancy between AN3 and ANT in leaf growth. Thus, these two proteins work in parallel and convergent pathways to regulate an unknown common target gene in cell proliferation (Figure 6). In our previous work, we suggested that *ORE15* delays the onset of senescence and regulates cell proliferation [25]. Our previous genetic analyses suggested that ORE15 primarily utilizes AN3/GIF1 in the regulation of leaf senescence. It has been reported that ANT plays a negative role in regulating leaf senescence, acting downstream of ARF2 [36], suggesting a possible regulatory relationship between AN3/GIF1 and ANT in leaf senescence.

It has also been suggested that cells in the leaf blade and petiole originate from a common proliferative region located at the leaf blade/petiole junction [37]. Our expression analysis showed strong expression of *ORE15* in the proximal part of the leaf blade and throughout the leaf petiole (Figure 1), partially overlapping with the expression of *AN3* and *ANT*. Strong expression of *AN3* and *GRF* family gene was also observed in the junction between the leaf blade and petiole in the current study (Figure 1) and in previous reports [9,15,37]. Our future work will aim to resolve the molecular mechanisms by which ORE15 coordinates the GRF–GIF pathway to regulate cell proliferation in the leaf blade and petiole, as well as leaf longevity.

## 4. Materials and Methods

### 4.1. Plant Material and Growth Conditions

The wild type *Arabidopsis thaliana* used in this study was Columbia-0 (Col-0). All mutants and transgenic plants in this study were originated from Col-0. The *ore15-2* and *ore15-1D* mutants were described previously [25]. *ore15-2* (SALK_029507) is a T-DNA insertional mutant, in which T-DNA was inserted in the first intron of the *ORE15* (At1g31040) and was obtained from the Salk collection. *ore15-1Dominant* (*ore15-1D*) was isolated from the T-DNA insert lines of the activation tagging vector, pSKI015 with tetrameric CaMV 35S enhancer repeats.

The *an3-4* and *ant-1* mutants were described previously [10,33]. *an3-4* has large deletionsin transcribed regions of the *AN3* (At5g26840) locus, which occurred by X-ray irradiation. The *AN3*promoter::β-glucuronidase (*GUS*) transgenic plants were kindly provided by G. Horiguchi [9]. Since *ant-1* homozygote is a female sterile plant and shows no visible defects at the early seedling stage, we isolated homozygotes by genomic PCR analysis to detect *ant-1*-associated nucleotide deletions as previously described [38]. *ant-1* has a 22 nucleotide deletion (from cDNA nucleotides 803 to 824) within the open reading frame of the *ANT* (At4g37750) locus, producing truncated ANT protein. For *ORE15* expression analysis using GUS assay, about 1.5 kb of DNA fragment of the upstream region of ORE15 gene were amplified from genomic DNA by PCR. The forward primer with *SacI* overhang and reverse primer with *SpeI* overhang at the 5′- end (Table S3) were used and then cloned into the pBluescript (-) vector. The *ORE15* promoter fragment was inserted by replacing the CaMV 35S promoter into pB2GW7 harboring *GUS* gene with *SacI* and *SpeI* restriction digestion. Transgenic plants were generated by the *Agrobacterium*-mediated floral dipping methods. Detailed information about these primers is listed in Supplementary Table S3. The plant seeds were surface sterilized, germinated on a Murashige and Skoog (MS) medium [39], and transferred to soil 3 weeks after sowing. Plants were grown at 22 °C under long daylight conditions (70–90 μE/m^2^·s, 16 h light/8 h dark cycle).

### 4.2. Histochemical Staining for GUS Activity and Anatomy of Leaves

GUS staining of transgenic plants harboring *ORE15*promoter::*GUS* or *AN3*promoter::*GUS* were performed by GUS assay method as described previously [40]. For anatomical analysis, samples were embedded in Technovit 7100 resin (Kulzer & Co. GmbH, Wehrheim, Germany) and examined as described previously [41]. Sectional slices were observed by a light microscopy (Axiskop2, Carl Zeiss, Jena, Germany). Rosette diameter was measured using the length from tip to tip of the longest rosette leaves from at least five plants at 35 DAS. Measurement of cell number and cell area for leaf growth analysis was performed as described previously [9,25]. At least seven mature third leaves were collected from WT and mutants on 21 DAS and used for measurement. To measure leaf area and width, the curling leaves were made flat by cutting the rolled margin of leaf blades as shown in Figure 3. Paradermal images of leaf cells from collected leaves were observed using a DIC optic by light microscopy, and number and area of leaf cells were determined as described previously [25]. Data was analyzed using the NIH IMAGE program imageJ (1.51j8, NIH, Bethesda, MD, USA) and statistically analyzed using Statistical Package for the Social Science (SPSS 13.0, SPSS Inc., Chicago, IL, USA) program.

### 4.3. RNA Isolation and Analysis of Gene Expression

Total RNA was isolated from the cell proliferating leaves—the third to fifth leaves of 13 DAS—using the RNeasy mini kit (Qiagen, Hilden, Germany) and cDNA was synthesized using the Reverse TraAce-a-First strand cDNA synthesis kit (TOYOBO, Tokyo, Japan) for reverse transcription. Primers used for qRT-PCR are listed in Supplementary Table S3. The qRT-PCR was performed using SYBR^®^ Premix Ex Taq^TM^ II (TAKARA, Otsu, Japan) with three technical replicates as described previously [25] and Bio-Rad CFX96^TM^ Real-Time System (Bio-Rad, Hercules, CA, USA). The relative expression level of each gene was calculated using the ΔΔCq method and the *TUB4* gene was used as a control [41].

## Supplementary Materials

Supplementary materials can be found at https://www.mdpi.com/1422-0067/21/1/241/s1. Figure S1: The relative alteration in cell number in a leaf from LOF and GOF single mutants and the combined mutants as in the corresponding control, Col-0 (WT), Figure S2: Distribution of DNA content in third leaves from Col-0 (WT), *ore15-2* mutant, and *ore15-1D* mutant plants during leaf development, Table S1: The area and width of mature 3rd leaves of Col-0 (WT), LOF and GOF single mutants, and the combined double mutants at 21 DAS, Table S2: The total number and area of palisade mesophyll cells in mature 3rd leaves from Col-0 (WT), LOF and GOF single mutants, and the combined double mutants at 21 DAS, Table S3: List of oligonucleotide primers used in this study.

## Abbreviations

Col-0	Columbia-0
ORE15	ORESARA15
AN3	ANGUSTIFOLIA3
ANT	AINTEGUMENTA
GIF	GRFINTERACTING FACTOR
GRF	GROWTH-REGULATING FACTOR
TCP	TEOSINTE BRANCHED 1, CYCLOIDEA, and PROLIFERATING CELL FACTORS 1 and 2
KRP1	Kip-related protein 1
WT	Wild type
LOF	Loss of function
GOF	Gain of function
SWP	STRUWWELPETER
