# Supplementary material for: ORESARA15 Acts Synergistically with ANGUSTIFOLIA3 and Separately from AINTEGUMENTA to Promote Cell Proliferation during Leaf Growth
Source: Int J Mol Sci. 2019 Dec 29;21(1):241. doi: 10.3390/ijms21010241 (PMC6981824; doi:10.3390/ijms21010241)
Supplement: Supplementary file 1 [file ijms-21-00241-s001.pdf]

Supplementary Materials and Methods

S1.1. DNA ploidy analysis

To analyze transition of cell cycle to endocycle, the 3rd leaves in mature stage were attached from Col-0 (WT), *ore15-2*, and *ore15-1D* on 21 DAS. DNA ploidy in leaves was measured by using nuclei staining method as described previously [39] with minor modification of propidium iodide (PI) instead of 4′,6-diamidino-2-phenylindole (DAPI). Ploidy were analyzed with the flow cytometer, Cytomics FC500 flow cytometer (Beckman Coulter, Brea, CA, USA).

Supplementary Figures

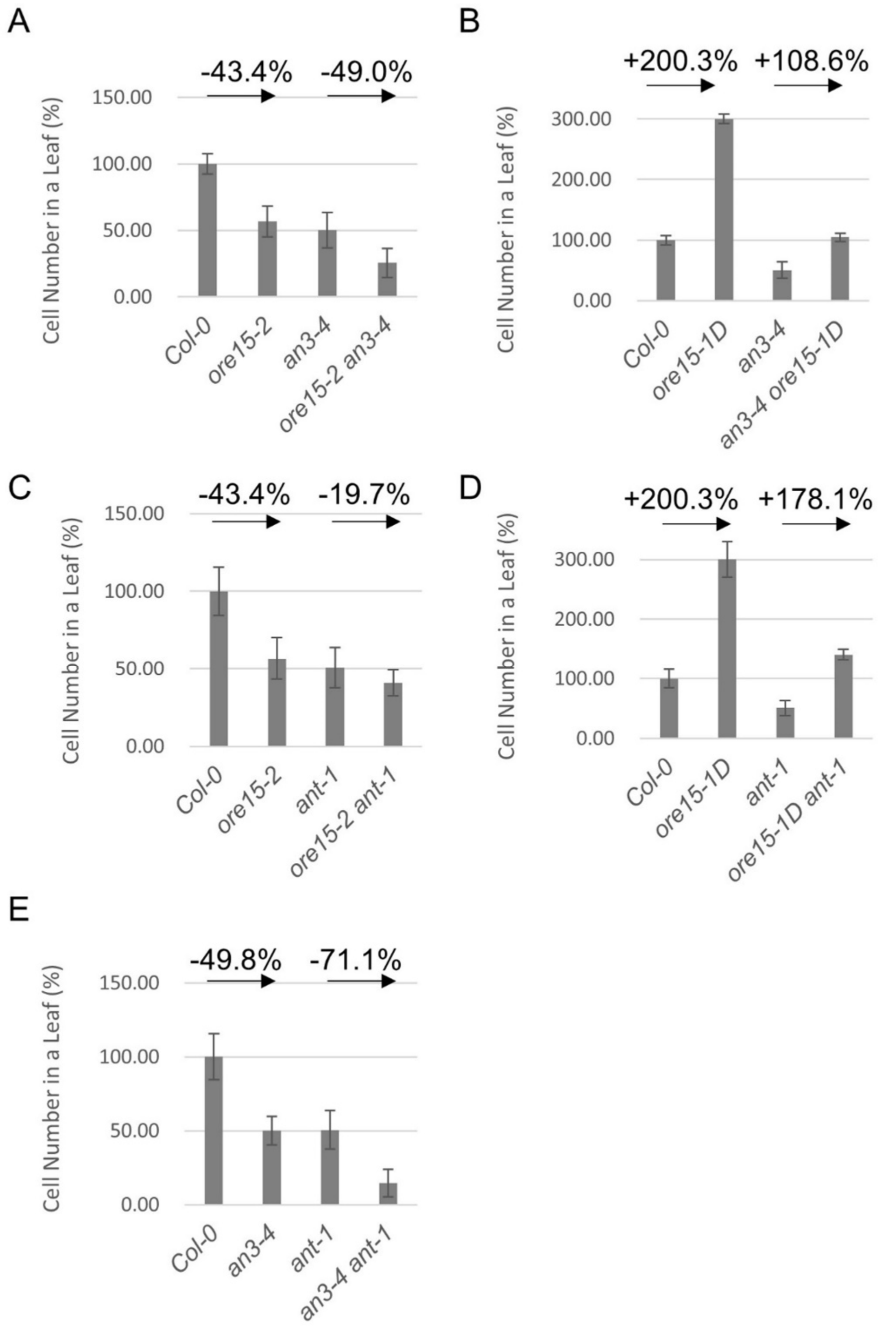

Figure S1. The relative alteration in cell number in a leaf from LOF and GOF single mutants and the combined mutants as in the corresponding control, Col-0 (WT). Numbers in plots indicate relative alteration of *ore15-2* in *ore15-2 an3-4* (A) and *ore15-2 ant-1* (C), relative alteration of *ore15-1D* in *an3-4 ore15-1D* (B) and *ore15-1D ant-1* (D), and relative alteration of *an3-4* in *an3-4 ant-1* (E) as in the corresponding controls, Col-0 (WT) and single mutants, *an3-4* (A, B) and *ant-1* (C,D,E).

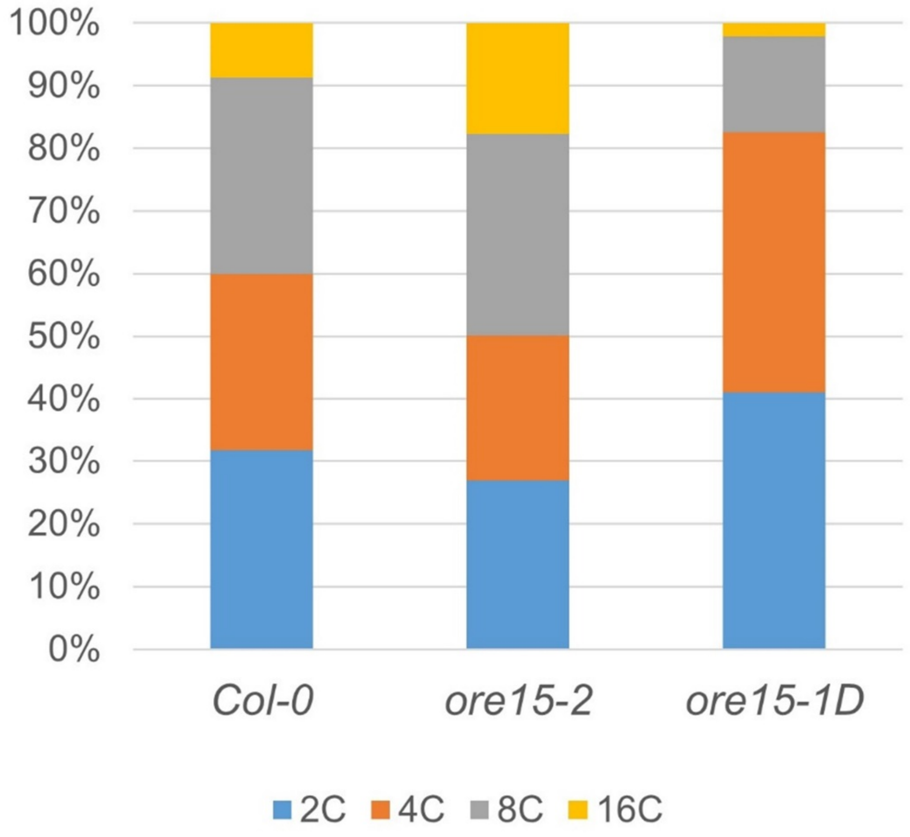

Figure S2. Distribution of DNA content in third leaves from Col-0 (WT), *ore15-2* mutant, and *ore15-1D* mutant plants during leaf development. Nuclei were isolated from five third leaves at the mature stage and analysed by flow cytometry.

Supplementary Tables

Table S1. The area and width of mature 3rd leaves of Col-0 (WT), LOF and GOF single mutants, and the combined double mutants at 21 DAS. Data are means ± SE (7 ≤ n ≤ 10).

	Actual		Relative value to WT (%)	
	Leaf Area (mm^2)	Leaf Width (mm)	Leaf Area	Leaf Width
Col-0 (WT)	44.3±2.3	6.7±0.2	100.0	100.0
ore15-2	26.7±4.5	4.9±0.6	60.2	72.0
ore15-1D	64.9±8.2	7.1±0.4	146.4	105.9
an3-4	24.7±3.4	4.7±0.4	55.8	69.1
ant-1	19.3±4.2	4.3±0.6	43.6	64.0
ANT OE-44	41.9±4.5	6.2±0.6	94.6	92.7
ore15-2 an3-4	13.1±2.8	2.9±0.5	29.5	43.3
ore15-2 ant-1	21.5±4.1	4.3±0.4	48.5	63.3
ore15-2 ANT OE-44	33.7±1.0	5.5±0.5	75.9	81.9
an3-4 ore15-1D	36.7±3.3	4.9±0.4	82.9	72.4
ore15-1D ant-1	39.3±4.9	5.7±0.5	88.7	84.2
ore15-1D ANT OE-44	44.9±6.6	6.7±0.6	101.2	99.7
an3-4 ant-1	8.7±2.5	2.6±0.7	19.7	38.7
an3-4 ANT OE-44	28.8±4.5	4.6±0.4	64.9	68.2

Table S2. The total number and area of palisade mesophyll cells in mature 3rd leaves from Col-0 (WT), LOF and GOF single mutants, and the combined double mutants at 21 DAS. Data are means ± SE (4≤ n ≤ 7).

	Actual		Relative value to WT (%)	
	Cell Number per a Leaf	Cell Area (μm^2)	Cell Number per a Leaf	Cell Area
Col-0 (WT)	43225.0±5275.1	989.8±75.9	100.0	100.0
ore15-2	24474.9±3301.5	1274.7±148.2	56.6	128.8
ore15-1D	129784.8±38581.9	523.9±40.2	300.3	52.9
an3-4	21683.9±2108.7	1384.2±185.7	50.2	139.8
ant-1	21899.5±2848.5	1065.2±103.7	50.7	107.6
ore15-2 an3-4	11061.8±1698.2	1385.7±152.5	25.6	140.0
ore15-2 ant-1	16426.2±1376.6	1472.2±280.7	38.0	148.7
an3-4 ore15-1D	44185.0±4467.0	1038.1±68.5	102.2	104.9
ore15-1D ant-1	60909.9±5454.9	872.8±107.4	140.9	88.2
an3-4 ant-1	6328.3±589.9	1661.3±358.3	14.6	167.9

Table S3. List of oligonucleotide primers used in this study.

Primer name	Sequence (5' to 3')	Description
ORE15_pro-F	GAGCTC GGAGCAAGCAGTAAAACGGGA	*ORE15* promoter cloning
ORE15_pro-R	GCTCTAGATTCTGTGGATTTTTATGGTAATTAAAG	
ant-1-F	CCTCAAACCAGAAACCAT	*ant-1* genotyping
ant-1-R	GGGCTCATGGATAAGCT	
ORE15-qRT-F	TCACTGTCTCCCTTCTCATCGC	qRT-PCR
ORE15-qRT-R	ACGTCGTGGTATACGTATCGTCTC	
AN3-qRT-F	TAA TGG CGG CTC GAT CTT CA	qRT-PCR
AN3-qRT-R	CTT CCC ACG GCC AAA ATC AT	
ANT-qRT-F	TCAATACCGAGGCGTTACAAGAC	qRT-PCR
ANT-qRT-R	TCGAGCAGCTTTCTCCTCCATATC	
CYCB1;1-qRT-F	ACCTCGCAGCTGTGGAATATGTG	qRT-PCR
CYCB1;1-qRT-R	CGGGTTTAGCTCGAATCGGACATGC	
CYCD3;1-qRT-F	CCTCAACAAATGCCACCGTCTC	qRT-PCR
CYCD3;1-qRT-R	AGGTACCCGACAAATCTTGAATCG	
GRF4-qRT-F	TTTCCTCATCACCAACCTTCTTGG	qRT-PCR
GRF4-qRT-R	CCCGCTACAACATCCCTTGAAC	
SWP-qRT-F	CAG AGT GAA TTA GTG AAG AG	qRT-PCR
SWP-qRT-R	CTC AAC ATA TTG AAT ATC CA	
TUB4-qRT-F	AGAGGTTGACGAGCAGATGA	qRT-PCR
TUB4-qRT-R	ACCAATGAAAGTAGACGCCA	
